# Polyamine Action under Metal/Metalloid Stress: Regulation of Biosynthesis, Metabolism, and Molecular Interactions

**DOI:** 10.3390/ijms20133215

**Published:** 2019-06-30

**Authors:** Mirza Hasanuzzaman, Haifa Abdulaziz S. Alhaithloul, Khursheda Parvin, M.H.M. Borhannuddin Bhuyan, Mohsin Tanveer, Sayed Mohammad Mohsin, Kamrun Nahar, Mona H. Soliman, Jubayer Al Mahmud, Masayuki Fujita

**Affiliations:** 1 Department of Agronomy, Faculty of Agriculture, Sher-e-Bangla Agricultural University, Dhaka 1207, Bangladesh; 2 Biology Department, College of Science, Jouf University, Sakaka 2014, Saudi Arabia; 3 Laboratory of Plant Stress Response, Department of Applied Biological Sciences, Faculty of Agriculture, Kagawa University, Kagawa 761-0795, Japan; 4 Department of Horticulture, Faculty of Agriculture, Sher-e-Bangla Agricultural University, Dhaka 1207, Bangladesh; 5 Citrus Research Station, Bangladesh Agricultural Research Institute, Jaintapur, Sylhet 3156, Bangladesh; 6 Stress Physiology Research Group, School of Land and Food, University of Tasmania, 7005 Hobart, Australia; 7 Department of Plant Pathology, Faculty of Agriculture, Sher-e-Bangla Agricultural University, Dhaka 1207, Bangladesh; 8 Department of Agricultural Botany, Faculty of Agriculture, Sher-e-Bangla Agricultural University, Dhaka 1207, Bangladesh; 9 Biology Department, Faculty of Science Yanbu, Taibah University, Al-Sharm, Yanbu El-Bahr, Yanbu 46429, Saudi Arabia; 10 Department of Botany and Microbiology, Faculty of Science, Cairo University, Giza 12613, Egypt; 11 Department of Agroforestry and Environmental Science, Faculty of Agriculture, Sher-e-Bangla Agricultural University, Dhaka 1207, Bangladesh

**Keywords:** Abiotic stress, toxic metal/metalloid(s), amino acids, stress signaling, reactive oxygen species, phytohormones

## Abstract

Polyamines (PAs) are found in all living organisms and serve many vital physiological processes. In plants, PAs are ubiquitous in plant growth, physiology, reproduction, and yield. In the last decades, PAs have been studied widely for exploring their function in conferring abiotic stresses (salt, drought, and metal/metalloid toxicity) tolerance. The role of PAs in enhancing antioxidant defense mechanism and subsequent oxidative stress tolerance in plants is well-evident. However, the enzymatic regulation in PAs biosynthesis and metabolism is still under research and widely variable under various stresses and plant types. Recently, exogenous use of PAs, such as putrescine, spermidine, and spermine, was found to play a vital role in enhancing stress tolerance traits in plants. Polyamines also interact with other molecules like phytohormones, nitric oxides, trace elements, and other signaling molecules to providing coordinating actions towards stress tolerance. Due to the rapid industrialization metal/metalloid(s) contamination in the soil and subsequent uptake and toxicity in plants causes the most significant yield loss in cultivated plants, which also hamper food security. Finding the ways in enhancing tolerance and remediation mechanism is one of the critical tasks for plant biologists. In this review, we will focus the recent update on the roles of PAs in conferring metal/metalloid(s) tolerance in plants.

## 1. Introduction

Polyamines (PAs) are ubiquitous, water-soluble polycations. They play a vital role in regulating plant physiology and development, as well as stress management [1,2] and newly invented plant biostimulant [3]. In all living cells, the chief PAs are found as diamine putrescine (Put), triamine spermidine (Spd), and tetramine spermine (Spm). They are positively charged and can bind to opposite charged molecules like DNA, RNA, adenosine triphosphate (ATP), proteins, and phospholipids [4]. Polyamines are also involved in regulatory processes, for example, plant growth, the division of the cell, cell differentiation, flowering, embryo development, senescence immunity, replication of DNA, gene regulation and nucleic acid synthesis [3,4]. In addition, PAs can scavenge the reactive oxygen species (ROS) [5] and play vital roles in regulating the plant defense response to diverse metal/metalloid(s) toxicity [1,6].

Metal/metalloid(s) contaminations of soils are considerably increased due to the different activities of human. For example, agricultural and industrial activities discharge a huge amount of toxic waste, therefore metal/metalloid(s) concentration increasing day by day [5]. For plant growth and productivity metal/metalloid(s) are very harmful, because they can induce toxicity in plant cells, thus can be carcinogenic for human if entered into the food web via plant. In addition, metals/metalloid(s) bind with proteins, which contain sulfhydryl groups, and therefore inhibit the activities of enzymes or alter protein structure. Under metal/metalloid(s) stress the toxic ions stimulate the formation of ROS in plants, hence, create oxidative stress by oxidation of macromolecules like protein, lipids, and DNA [7]. Several reports suggested that excess metal/metalloid(s) can be an obstacle with the metabolism of PAs [6,8]. Groppa et al. [6] reported that the metabolisms of PAs are influenced by the application of Cd^2+^ or Cu^2+^ notably in wheat or sunflower leaf. In a recent study, Tajti et al. [9] reported that PAs pretreatment could accelerate metal chelation, maintain hormonal balance, enhance an antioxidant defense, and conferred cadmium (Cd) tolerance in wheat.

The exogenous application of PAs regulates the antioxidative mechanisms in plants under metal/metalloid(s) stress to mitigate the overproduction of ROS [8]. Under Cd stress, the exogenous application of Put and/or sodium nitroprusside (SNP, NO donor) scavenged ROS by improving the activity of enzymes (superoxide dismutase, SOD; catalase, CAT; ascorbate peroxidase, APX; monodehydroascorbate reductase, MDHAR; dehydroascorbate reductase, DHAR; glutathione reductase, GR; glutathione *S*-transferase, GST; and glutathione peroxidase, GPX) and nonenzymatic antioxidants (ascorbate, AsA and glutathione, GSH) [1]. The exogenous application of Spd improved Cd tolerance in *Boehmeria nivea* L. plant [10]. Rady and Hemida [11] found that the seed priming with Spm or Spd enhanced the Cd tolerance in wheat seedlings. Taie et al. [12] also reported that seed soaking or foliar application of Spm, Spd or Put considerably improved wheat plant growth and yield under Cd and Pb stress by increasing tolerance mechanisms. Benavides et al. [5] investigated the effect of Cd and Cu on wheat and sunflower plants and found that membrane fluidity altered in plant leaves and roots during germination and seedling stage, but pre-treated plants with PAs avoided that alteration. Therefore, PAs can enhance the tolerance in plants to sustain under metal/metalloid(s) stress condition. Hence, in this review, we have summarized the current knowledge concerning PAs (biosynthesis, metabolism, and molecular interaction) in the plant under metal/metalloid(s) stress. Thus, the objectives of this review to explain the possible roles of PAs improving plant tolerance in relation to antioxidant defense, metal chelation and interaction with other plant molecules under metal/metalloid(s) stress.

## 2. Polyamines Biosynthesis and Metabolism

**Figure 1:**
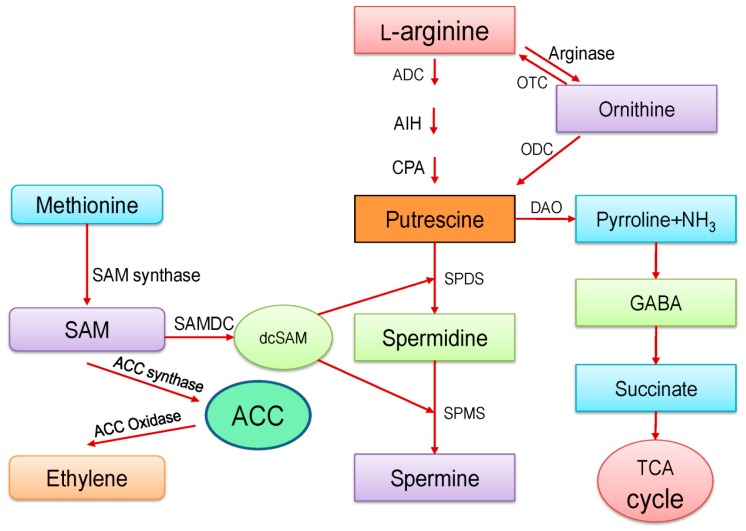
Biosynthesis pathway of polyamines in plants. ADC: arginine decarboxylase; AIH: agmatine iminohydrolase; CPA: N-carbamoylputrescine amidohydrolase; SPDS: spermidine synthase; SPMS: spermine synthase; OTC: ornithine transcarbamoylase; ODC: ornithine decarboxylase; DAO: diamine oxidase; GABA: γ -aminobutyric acid; SAM: *S*-adenosylmethionine; SAMDC: S-adenosylmethionine decarboxylase; dcSAM: decarboxylated *S*-adenosylmethionine; ACC synthase: 1-aminocyclopropane-1-carboxylic-acid synthase. Arrows represent the synthesis/conversion.

In plants, briefly, PAs biosynthesis starts with the decarboxylation of either L-arginine (Arg) directly (by arginine decarboxylase, ADC) or from ortinine (by ortinine decarboxylase: ODC) indirectly (Figure 1). The ADC pathway directly produced Put by the chronological action of three enzymes; ADC, agmatine iminohydrolase (AIH), and N-carbamoylputrescine amidohydrolase (CPA), whereas in the ODC pathway, Arg first converts into ornithine by the action of arginase and then ornithine converts into Put. Production of orthinine from Arg depends on arginase enzyme activity. After carboxylation steps, the high molecular weight of PAs; Spd and Spm are produced from Put by the sequential addition of aminopropyl groups to Put and Spd and by the action of Spd synthase (SPDS) and Spm synthase (SPMS) enzymes, respectively. Moreover, decarboxylated *S*-adenosylmethionine (dcSAM) further regulates the activity of SPDS and SPMS to produce Spd and Spm. On the contrary, dcSAM is generated by the decarboxylation of *S*-adenosylmethionine (SAM). Besides, SAM also acts as a precursor of ethylene (ET) production and converts into ET by 1-aminocyclopropane-1-carboxylic-acid synthase (ACS) and oxidase enzyme. Besides catabolism of Put and Spd yields γ-aminobutyric acid (GABA) via pyrroline. Yet, GABA from this step converts into succinate, which further enters in the TCA cycle (Figure 1).

In different plant species, genes for PAs biosynthesis enzymes have been identified, characterized, and cloned; however, genes encoding sequences for all the above-mentioned enzymatic activities, with the exception of ODC, have only been characterized in *Arabidopsis* [13]. In *Arabidopsis*, no ODC activity has been detected so far [14], while two genes for ADC activity (*ADC1* and *ADC2*) and one gene for AIH and one gene for CPA have been identified. According to Alcázar et al. [15], to promote Put accumulation in *Arabidopsis* the overexpression of homologous *ADC2* is sufficient, which suggests that for Put biosynthesis ADC is the limiting step in plants. There are two genes for SPDS (*SPDS 1* and *SPDS 2*), two genes for SPMS (*SPS* and *ACL5*) and four genes for SAM decarboxylation (*SAMDC1*, *SAMDC2*, *SAMDC3*, and *SAMDC4*) have been identified and characterized to examine their role in PAs biosynthesis [16,17,18]. It was reported that PAs produced from different pathways appeared to have a differential role in plant development. For example, PAs produced via ODC pathway play a role in floral development, while PAs generated via ADC pathway involved in vegetative development [19]. Moreover, it has also been suggested that ODC is related to the regulation of cell division/proliferation, while ADC involved in the stressed tissue and/or extension of the cell [19].

Under metal/metalloid(s) stress conditions, PAs accumulation increased by the stimulation of the different steps involved in the PAs biosynthesis process, not by triggering high molecular weight PAs, i.e., Spd or Spm [20]. For instance, under Cd stress, Kuthanová et al. [21] showed increased PAs accumulation mainly due to the high Put accumulation and diamine oxidase (DAO) activity. However, Choudhary and Singh [22] did not find any effects of Cd stress on DAO, despite high PAs accumulation in mung bean. In another study, Groppa et al. [6] showed that high PAs accumulation under Cd stress was due to high ADC and ODC activity in wheat while under Cu stress; only ODC was noted as the main enzyme involved in the higher Put synthesis. Similarly, in maize under Pb stress, a higher concentration of total PAs has been noted as compared with control, and this was associated with the higher Put synthesis.

## 3. Polyamine-Induced Metal/Metalloids Tolerance in Plants

**Figure 2:**
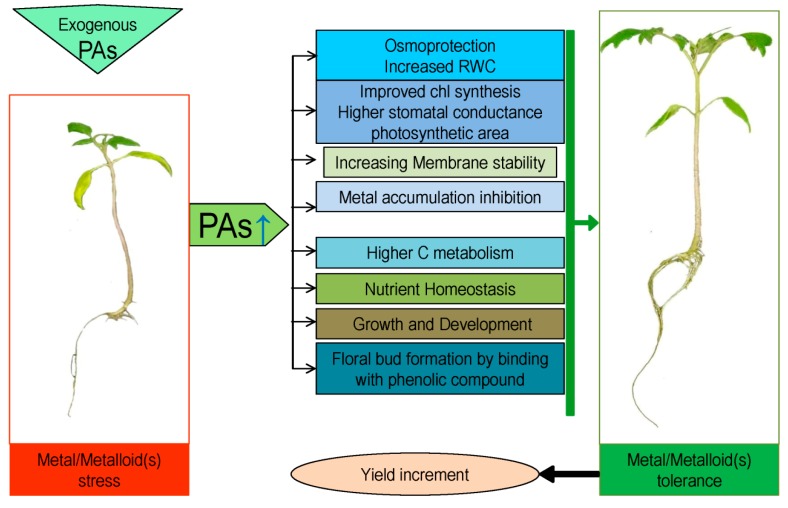
Metal/metalloid(s) stress tolerance by polyamines (PAs). Blue arrow represents the upregulation of PAs accumulation, black arrows represent the enhancement and green arrow represent the tolerance.

No plants want to die, and thus try to survive under stress by modulating self-defense mechanisms. As part of this, plants often endogenously synthesize PAs. In previous sections, we stated that PAs retrieve abiotic stresses including metal/metalloid(s). In this section, we will discuss how PAs induce metal/metalloid(s) stress in plants. As sessile organism plants have to survive to tolerate the stress. Therefore, plants have evolved some protective mechanisms against metal/metalloid(s)-induced stresses, such as elimination, metal chelation, and compartmentalization [1] (Table 1; Figure 2). Antioxidant defense system present in a plant cell is very efficient, through which plants cope with metal-induced oxidative stress. Polyamines have an intimate relationship with this antioxidant defense. Plant synthesizes PAs endogenously, which further enhanced antioxidant defense mechanisms, including energizing antioxidants, ROS scavenging, metal chelation, and membrane stability [1,23]. Upon stress, PAs work as signaling molecules and consequently regulates ion homeostasis and control ion transportation through interacting with ion channels [24]. Thus, PAs play not only vital roles in normal plant developmental and physiological processes but also have active participation in abiotic stress tolerance [25]. Therefore, stress affected plants alleviate the adverse effect of stress through the expression of PAs biosynthetic genes as well as improving PAs biosynthesis [26].

Kinnow mandarin (KM) grafted plants showed increased free, soluble-conjugated (PS conjugated PAs) and insoluble-bound PAs (PIS-bound PAs) content in leaves, when exposed to Cr-toxicity [27]. Therefore, increased PAs might be due to the stimulated activity of PAs anabolism enzymes (such as ADC, ODC, SAMDC, and SPDS) with lower activity of catabolic enzymes (polyamine oxidase: PAO and DAO) in plant leaves upon exposure to metal toxicity [27].

Howladar et al. [30] found an enhanced accumulation of endogenous Spd and Spm in Cd affected wheat, thus indicating a crucial plant-induced mechanism to avoid metal/metalloid(s) stress. Upon metal stress, the plant can regulate the accumulation and translocation with the increased endogenous PAs, where exogenous PAs stimulates this metal tolerance mechanism [1]. They studied the Cd-stressed mung bean response followed by PAs induced protection. Here, they used Put (0.2 mM) against 1.5 mM Cd exposure. They found a lower biological accumulation coefficient (BAC) and translocation factor (TF), biological concentration factor (BCF), the value in mung bean after application of Put (0.2 mM) in 1.5 mM Cd-exposed seedlings, which resulted in lower Cd concentration in both root and shoot. Hence, they indicated the PA-induced lower metal/metalloid(s) accumulation as well as translocation from cell to cell. This inhibitory mechanism of PAs on metal/metalloid(s) uptake causes metal/metalloid(s) tolerance in plants. Not only this, but PAs also enhances the production and accumulation of phytochelatins (PC), which efficiently binds metal/metalloid(s) and this is considered as one of the most efficient metal/metalloid(s) detoxification strategy. Here, the presence of toxic metal/metalloid(s) induces the activity of PC synthesis by using the GSH as a substrate to form PCs, which bind metal ions and transport into the vacuole for safely carrying metal away from plant cells. According to research finding, PAs are able to increase GSH content and subsequently the PCs content under metal/metalloid(s) stress [1]. From a recent study of Howladar et al. [30], it is also clear that increased GSH content along with higher endogenous Spd and Spm in Si treated Cd stressed wheat plants provided tolerance against metal/metalloid(s) toxicity. Therefore, GSH itself involves metal/metalloid(s) chelation and detoxification [40]. Thus, maintaining higher GSH content is an essential mechanism for metal/metalloid(s) tolerance in plants. Many research findings confirmed about the PAs-induced elevated GSH content under metal/metalloid(s) stress as a tolerance mechanism [1,11,30]. Afterward, metal/metalloid(s) detoxification in plant showed improved plant growth osmotic status and Chl synthesis as signs of tolerance (Table 1). Nahar et al. [1] also found restoration of plant growth, relative water content (RWC), and Chl synthesis with higher Pro accumulation after Put treatment in Cd affected seedlings. They concluded that Put induced Pro accumulation at a higher amount might give the osmotic protection to plant. This greater Pro accumulation in the metal affected plant may be due to higher Pro synthesis for enhancing tolerance [41]. Other scientists have also reported about the PAs induced enhanced photosynthetic activity and functioning of photosynthetic apparatus under stress exposure although [42,43,44]. Exogenous use of PAs as seed treatment also induced better performance in *T. aestivum* regarding Cd stress [31]. Where, 0.25 mM Spm, 0.50 mM Spd, and 1.0 mM Put was used to improve growth and yield under 2.0 mM Cd exposure. They concluded that 1.0 mM Put pretreatment was best regarding tolerance response compared to other PAs with greatest RWC, membrane stability, photosynthetic pigment synthesis, mineral nutrients content and osmoprotectant concentration [31]. Ghabriche et al. [45] explained that PAs (Put, Spd, and Spm) brought a reduction of Cd-induced damage in *Inula crithmoides* by stabilizing cellular structure through modulation of nutrition and ammonium/nitrate ratio.

## 4. Polyamine-Induced Antioxidant Defense in Plants under Metal/Metalloid(S) Toxicity

**Figure 3:**
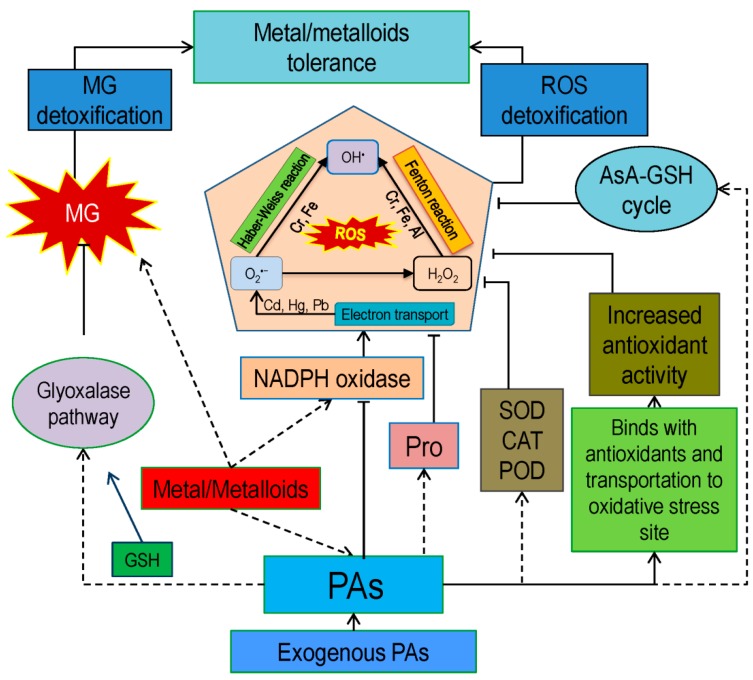
Polyamine-induced antioxidant defense and glyoxalase system under metal/ metalloid(s) stress. Dotted arrows represent stimulation/upregulation, solid arrows represent conversion/synthesis and “T” bar represent scavenging/detoxification.

Like other abiotic stresses, metal/metalloid(s) also impose oxidative stress by generating excessive ROS (singlet oxygen: ^1^O_2_; superoxide anion: O_2_^•−^; hydrogen peroxide: H_2_O_2_; and hydroxyl radical: OH^•^), imbalance ROS homeostasis, and subsequent ROS−mediated damage of biomolecules such as protein, lipid, DNA, etc. [1]. Although plants are efficient with antioxidant defense, which fight back to scavenge ROS through both nonenzymatic and enzymatic antioxidants [1] (Figure 3), the efficiency of these processes may decrease gradually. At this point, PAs can actively participate in mitigating oxidative damage, through ROS scavenging [46,47]. Sometimes, H_2_O_2_ can be produced from the activities of DAO and PAO enzymes to degrade PAs, and thus causes stimulation of the antioxidative defense system. Moreover, PAs possess both anionic and cationic binding sites, which bestow radical scavenging and antioxidant properties, ultimately inhibit lipid peroxidation and oxidative reactions [48]. Polyamines contribute to binding the anions (phospholipid membranes and nucleic acids) in cells particularly prone to oxidations, whereas the cations efficiently prevent the generation of site-specific ROS, such as OH^•^ and ^1^O_2_ [49]. Polyamines also protect the membranes from oxidative attack by forming a complex with phospholipid and Fe^2+^ that can prevent the Fe^2+^ auto-oxidation [50]. Yu et al. [29] found that Put play a protective role in preventing Al-induced oxidative stress, where H_2_O_2_-generating both enzymes named CW-PAO (cell wall-PAO) and plasma membrane NADPH oxidase were inhibited. Moreover, Phenylpropanoid-PAs conjugates also react with reactive nitrogen species (RNS) and ROS and modulate the activities of enzymatic antioxidants (SOD; peroxidase: POD; and CAT), enhance ROS detoxification and subsequent inhibition of lipid peroxidation in metal-stressed seedlings [27] (Table 2).

Exogenous PAs stimulated the SOD, POD and CAT activities under stresses [1,47,51]. Thus, there is a positive correlation between PAs and antioxidants for ROS detoxification. Nahar et al. [1] showed the protective role of exogenous Put (0.2 mM) under Cd (1.5 mM) toxicity in Mung bean. Their research group found that the application of Put positively modulated endogenous PAs (Put, Spd, Spm) accumulation and this free PAs contributed to the reduction of oxidative damage through enhancing the antioxidant defense system. They also found, both enzymatic (SOD, CAT, APX, DHAR, GR, GST, and GPX) and nonenzymatic (AsA and GSH) antioxidants become up stimulated. Consequently, excessively produced ROS dismutated to nontoxic compound and plant got relief from oxidative stress by showing lower ROS generation compared to stressed plants. Together with this, they also found that PAs detoxified the toxic methylglyoxal (MG, another vital cytotoxic compound, which also causes oxidative stress) by enhancing the glyoxalase system through the upregulation of both glyoxalase I (Gly I) and glyoxalase II (Gly II) enzymes. Thus, PAs, including Put, relieve oxidative stress directly and indirectly.

Polyamines like Spd increase plant tolerance to metal toxicity by enhancing AsA and GSH pool and subsequently balancing redox homeostasis. For example, Spd protects *R. sativus* from negative impacts of Cr [34]. Yang et al. [36] demonstrated the PAs involvement in Cd affected *Potamogeton crispus* plant. They found that upon Cd exposure endogenous free PAs content increased along with PIs bond Put content. Afterward, AsA and GSH contents were also increased, which further contributed to ROS detoxification. Therefore, in light of the above discussion, it is clear that PAs modulate the antioxidant defense system under metal/metalloid(s) toxicity for inhibiting ROS induced oxidative damages.

## 5. Polyamine-Induced Metal/Metalloid(s) Chelation and Phytoremediation in Plants

**Figure 4:**
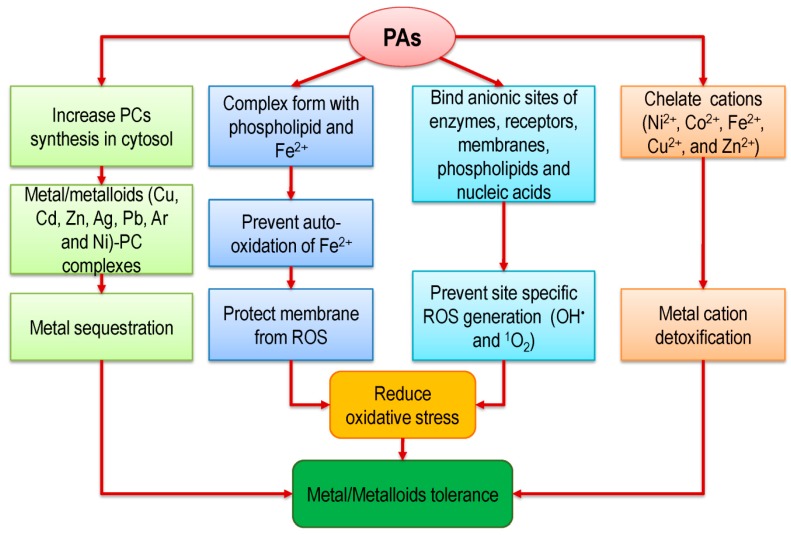
Proposed mechanisms of PA-induced metal chelation in plants.

Under metal/metalloid(s) stress the common defense mechanisms evolved by plants are exclusion, complexation, compartmentalization, and metal-binding protein synthesis and ion chelation [52,53,54]. Among them, the most important mechanism is chelation, which maintains the free metal ion concentration in the cytoplasm via detoxification. Chelation can be accomplished by thiol compounds that contain γ-Glu-Cys-Gly, phytochelatins (PCs), metallothioneins (MTs), and sulfhydryl/thiol groups (tripeptide glutathione, and GSH)) and also non-thiol compounds, for example, organic acids and amino acids [53,55] (Figure 4). Amino acids and their derivatives play a vital role in chelation of different metal/metalloid(s) and tolerance in plants. However, efficient mechanisms for chelation by amino acids are not so conclusive [40]. Amino acid derivatives, PAs are recommended as one of the vital metal/metalloid(s) chelators. For example, after application of PAs in European pear (transgenic line) the chelation of metals (Zn, Cd, and Pb) increased, thus enhanced metal tolerance [56].

Polyamines are cationic (Put^2+^, Spd^3+^, and Spm^4+^), and hence have the ability to bind nucleic acids [25,57]. As cations, PAs interact with anions, and they can imitate and compete for the same binding sites with Mg^2+^ and Ca^2+^ on enzymes, receptors, and membranes. For cellular protection, PAs also bind some cations including Cu^2+^ and Fe^3+^ [49]. However, the presence of several complex-forming groups in the same molecule regulates and promotes chelation effects. Therefore, PAs with a high number of N-groups act as a stronger chelator or enhance chelation mechanism [1,49]. The synthesis of PCs under metal stress is another vital strategy of plants to tolerate metal/metalloid(s) toxicity at different developmental stages, which is influenced by PAs [58]. Several researchers found that exogenous PAs application increased the cellular PCs content [1,34]. While Groppa et al. [39] found that there are no effects of PAs on PCs biosynthesis, Nahar et al. [1] found that, Put application increased the content of PCs in the Cd-affected mung bean seedlings, which indicate the interaction of PAs for Cd chelation. Similarly, Spd application in *Raphanus sativus* L. plant under Cr-stress increased PCs content [34]. Contrary, Pál et al. [58] found that pre-treatment of Put prevented the synthesis of PCs in rice at the molecular and gene expression levels.

If we think from an economic point of view, phytoremediation can be useful for reducing risk of metal/metalloid(s), gradually improves soil quality, phytoextraction of high market value metals [59]. Moreover, using fast-growing timber trees, the toxic metal/metalloid(s) could be trapped in the wood [60]. Thus, the success of phytoremediation mainly depends on shoot metal concentration. As previously described, PAs with a high number of N-groups act as a stronger chelator or enhance chelation mechanism. Polyamines are already used as an adsorbent to purify water. Keymirov [61] reported that natural montmorillonite modified by PAs can remove metal/metalloid(s) ions from water in the series Cu(II) > Ni(II) > Zn(II) > Cd(II). However, in plants, a variety of molecules control and regulate the journey of metal/metalloid(s) ions from the soil solution to the vacuoles, where some perform cross-membrane transport when others functions in their complexation and subsequent sequestration. There are contradictory reports showing the PAs induced phytoremediation regarding the metal/metalloid(s) uptake and translocation but most of the researchers’ affirmed that the PAs increase formation of PCs and sequestering metal/metalloid(s) in vacuoles. Previous reports suggested that in Cd-stressed mung bean seedlings, the root Cd content was much higher than the shoot Cd content, while BCF, TF, and BAC also increased [62]. On the other hand, Shevyakova et al. [63] reported that PAs treatment increased the capability of Ni accumulation by 2–3 times and reduced Ni toxicity by chelation. Again Pál et al. [58] reported that PAs application increased shoot Cd content by 5-fold mainly through chelation. Polyamines also induce the formation of PCs [58,62], which further accelerate the phytoremediation process. Therefore, in light of the previous discussion, it can be portrayed that PAs induced direct metal/metalloid(s) chelation or indirect chelation through GSH dependent PCs are the main valuables for the PAs modulated phytoremediation process. Thus, nevertheless PAs reduce metal/metalloid(s) translocation, yet they can be a potential candidate enhancing the phytoremediation process.

Besides, PAs induced metal/metalloid(s) chelation opens a new portal to enrich our foods with various micronutrients. Soudek et al. [64] reported that vegetables grown on micronutrients (Fe or Zn) enriched medium might exceed their uptake and cause adverse health effects for consumers. Here, PAs like Put is able to increase or decrease metal/metalloid(s) micronutrient uptake depending on the plant species. On the other hand, their findings also suggested that the consumption of these enriched vegetables could be a solution for nutrient deficiency in our diet. Although these studies are still in preliminary stages, it is expected that more interesting result will come in the future. Therefore, further research should be highlighted on the PAs induced metal/metalloid(s) transportation from root to shoot, PAs induced GSH content upregulation and PCs synthesis to upscaled the phytoremediation process in plants. Moreover, PAs induced metal/metalloid(s) chelation based food fortification might be a new focused area in the future.

## 6. Interaction of Polyamines with other Molecules in Conferring Metal/Metalloids Tolerance in Plants

**Figure 5:**
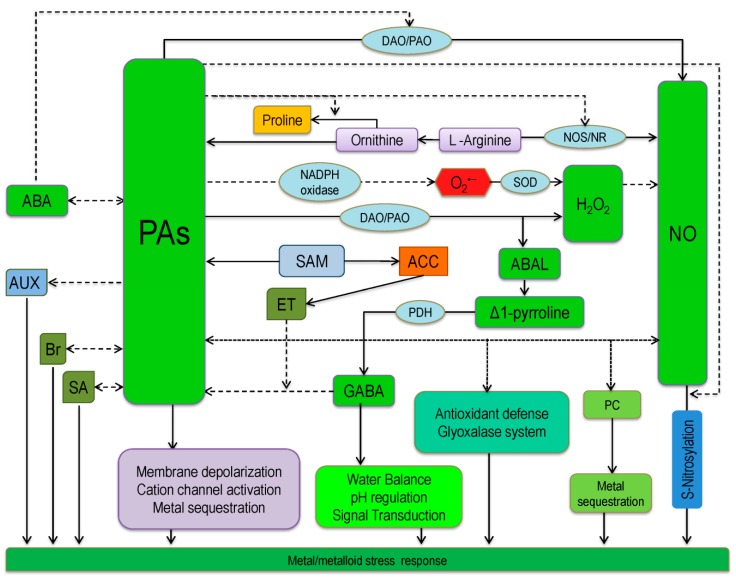
Interaction of polyamines with other molecules conferring metal/metalloid stress in plants. Dotted arrows represent stimulation/upregulation and solid arrows represent conversion/synthesis.

In the previous sections, we discussed the metabolism of PAs in the plant. We also highlighted PAs-assisted metal/metalloid(s) tolerance and the antioxidant defense under metal/metalloid(s) toxicity along with metal/metalloid(s) chelation and sequestration in plants. Besides, these PAs play interacting role with other biomolecules to retrieve of metal/metalloid(s) stress, including interaction with osmolyte (proline: Pro; glycine betaine: GB), compatible solute (GABA), macromolecules (DNA and RNA) which contribute in reducing ROS generation, scavenging and signaling, enhancing antioxidant metabolisms, signaling role with other signaling molecules and regulating ion channels ([24,65]; Figure 5). Moreover, it was reported that increased PAs contents regulate the gene expression of various plant secondary metabolites and signaling molecules. Hence, in this section, we will summarize the cross-talk and interaction of PAs with biomolecules for metal/metalloid(s) tolerance.

### 6.1. Nitric Oxide and Polyamines Cross-talk and the Reversal of Metal Phytotoxicity

Being gaseous with diffusible nature, NO regulates various physiological and developmental processes as an intra- and intercellular messenger along with activation of plant stress responses. Alike NO, PAs also play diverse roles in regulating many physiological processes, including organogenesis, embryogenesis, flower and fruit development, and senescence mechanism [25]. Therefore, there might be a link between NO and PAs metabolism, which is partially reported previously. In the biosynthetic pathway, PAs and NO both share Arg as a common precursor [66], hence PAs are supposed to modulate the Arg-linked NO synthase (NOS) and nitrate reductase (NR) pathways [67]. Rapid NO accumulation without a lag phase was reported in *Arabidopsis* after Spd and Spm treatments, whereas Put had little or no effect [68]. Opposite to this finding, PA-induced NO production was observed in *Arabidopsis* NR impaired double mutant nia1nia2, showing NR is not the only contributor to NO production. Yet, experimental evidence on PA-induced NO biosynthesis proposed new insights that the PAs can be directly converted to NO with the activity of PAO and DAO [15]. Therefore, NO attributes to many functions on PA-mediated metal stress responses. In addition, NO and PAs have some common roles under stress conditions, and hence they linked each other, conferring the metal/metalloid(s) stress [1]. Some researchers hypothesized that NO directly or indirectly seal the slits of PAs induced physiological effects to mitigate metal/metalloid(s) stress [1,69]. Exogenously applied Put and/or SNP (NO donor) increased endogenous PAs (Put, Spd, and Spm) and NO content; where MG detoxification was observed by the improved glyoxalase system along with better physiology and growth [1]. They also found higher PC contents in Cd-affected seedlings, which suggested the role of both PAs and NO in upregulating the PC biosynthesis along with Cd sequestration. In this study combined application of PAs and NO showed better effects, which showed the possible cross-talk between NO and PAs to confer Cd-tolerance.

### 6.2. Reactive Oxygen Species and Polyamines Interaction and the Reversal of Metal Phytotoxicity

Another vital double-edged aspect is that PAs can be sources for ROS production as well as efficient ROS scavengers, and also play a role in balancing redox homeostasis in plant tissue [70]. During PAs catabolism two PAs catabolizing enzymes, PAO and DAO modulate the endogenous PAs levels, where, H_2_O_2_ is liberated in the apoplast and peroxisomes [71]. However, as a signal transduction molecule H_2_O_2_ is able to modulate various physiological and biochemical processes, including influencing ion channels for stomatal regulation, activating the stress response via the MAPK cascade, etc. [72] while activating the antioxidative system under metal/metalloid stress conditions [29]. Hence, PAs, especially Spm enhance the activity of NADPH-oxidase and produce O_2_^•−^. Further, O_2_^•−^ turns into H_2_O_2_ by the spontaneous involvement of SOD. The ratio of O_2_^•−^ and H_2_O_2_ is a vital signal to the transcription process [73], hence can mediate PAs induced plant adaptation to metal/metalloid(s) stress [29,74].

### 6.3. Interaction of Polyamines with GABA and Pro Conferring Metal/Metalloid(s) Stress

The role of GABA is well established as a neurotransmitter in animal cells. In plants, increases in GABA level are suggested to contribute to stress protection through the regulation of cellular pH, acting as osmoregulator or as signaling molecules. In the PAs metabolic pathway 4-aminobutanal (ABAL), H_2_O_2_, and NH_3_ are produced as a byproduct with the activity of PAO and DAO [75]. Produced ABAL is then instinctively converted to form Δ^1^-pyrroline, which further converted into GABA by pyrroline dehydrogenase (PDH). Hence, enhanced PAs metabolism may lead to increased GABA accumulation, as well as stress tolerance [76]. On the other hand, GABA accumulation has been suggested to reduce the oxidative damage caused by ROS, leading to improved tolerance to oxidative stresses [77]. These observations suggest the intrinsic relationship between PAs and GABA during abiotic stress. The exogenous GABA application also influences PAs metabolism, and hence confers stress tolerance. Increased metal chelation and activation of antioxidant defense and glyoxalase systems to alleviate the oxidative damage from ROS and MG by exogenous GABA application has been reported in Cr-exposed Brassica seedlings [78], and both Al and acidity stressed barley seedlings [79]. Moreover, GABA activates multiple mechanisms involved in signaling cascades, regulation of protein degradation, hormone biosynthesis, ROS metabolism and PAs metabolism in response to stress. Shi et al. [80] reported that exogenous GABA influences the gene expression of genes involved in PAs biosynthesis in response to abiotic stress. Furthermore, GABA reduced the DAO gene expression as well as DAO activity and alleviated PAs metabolism, especially reduced Put catabolism to GABA [81,82]. These findings speculated the role of GABA as an alternative modulator of tolerance by regulation of PAs metabolism in plants and suggested that under stress situation, GABA increases PAs levels by suppressing its formation. However, although several reports under abiotic stress discussed the interacting role of PAs and GABA, still there is a shortage of literature evaluating the cross-talk between PAs and GABA for conferring abiotic stress tolerance especially metal/metalloid(s) stress. Therefore, the roles for GABA under metal/metalloid(s) stress need to be defined, especially the PAs and GABA regulatory mechanisms. Further studies are also needed by combining genomic and metabolomic approaches to clarify the interconnection between GABA and PAs, together with their secondary metabolites in conferring metal/metalloid(s) stress tolerance in plants.

The cross-talk between PAs and Pro in regulating stress tolerance has also been widely described. Proline, alanine (Ala), glutamine (Glu), and GABA are all GABA shunt-related metabolites that accumulate in response to ROS production in plants [83,84]. The exogenous application of PAs induced stress tolerance and Pro accumulation in mung bean exposed to Cd and Al toxicity [1,62,85]. Cvikrová et al. [86] analyzed P5CSF129A transgenic tobacco, which accumulated higher Pro, Put and Spd levels, showing dehydration tolerance and recovery capacity than wild type. Therefore, new studies combining genomic and metabolomic approaches are needed to clarify, how PAs increases Pro metabolism, and whether other metabolites interconnected, regulating osmolyte synthesis for conferring stress tolerance under metal/metalloid(s) toxicity.

### 6.4. Interaction of Plant Hormones and Other Hormone-Like Protective Molecules with Polyamines

Although the PAs and phytohormones (auxins: AUX; cytokinins: CK; gibberellins: GA; abscisic acid: ABA; brassinosteroids: Br; ET; jasmonic acids: JA; salicylic acid: SA) are interrelated, and their activity and interactions are not yet elucidated fully. Agami [87] found the contribution of indole acetic acid (IAA) in Spd induced Cu-stress tolerance in *T. aestivum*. Previously we discussed the interacting role of PAs with NO. Here, it is worthy of mentioning that NO take part as an intermediate signaling molecule in AUX, CK, ABA, and ET signaling. Transgenic plants overexpressing *ADC2* showed the lower content of GA1, GA4, and GA9, and reduced the *AtGA20ox1*, *AtGA3ox1* and *AtGA3ox3* transcripts expressions [15], suggesting that Put accumulation inhibits GA synthesis. Similarly, the mutant *SAMDC4* exhibited hyposensitivity to exogenous AUX and hypersensitivity to CK treatments. These findings suggested that PAs increases AUX sensitivity, and reduce CK biosynthesis or signaling [88].

There are controversies between the interaction of PAs and ET, whether the relationship is antagonistic or synergistic. Yin et al. [89] reported that synergistic interactions were exhibited between PAs and ET, where they found decreased 1-aminocyclopropane-1-carboxylic-acid (ACC) levels with the increase of PAs concentration, hence, the leaf senescence slowed down. Some researchers termed the relationship as antagonistic as ET enhance senescence while PAs inhibit [90]. Yin et al. [89] observed ACS activity is inhibited by Put, which lowered ET production and eventually alleviated Al-induced root inhibition. Hence, two pathways were not strictly antagonistic. Yet again, NO can also modulate ET biosynthesis and its signaling [91]. Through *S*-nitrosylation, NO can change the activity of methionine adenosyltransferase (MAT), and hence causes a reduced formation SAM, which is obvious for ACC generation and ET production [92,93]. Under metal/metalloid(s) toxicity, especially during Cd-exposure, Cd modulated the genes encoding the proteins; those are involved in ET and PAs metabolism, as well as NO generation, Mitogen-activated protein kinases (MAPKs) cascades and regulate other gene expressions in *G. max* seedlings, including *ACS, SAMDC*, *MAPK*, and *MAPKK2*, and *DOF1*, *MYBZ2*, and *bZIP62* transcription factors in Cd tolerance [94].

As ABA is very much linked to dehydration-induced by metal/metalloid(s) stress [1], hence, ABA modulated PAs metabolism during the transcriptional stage, and regulate the expression of PAs biosynthesis pathway genes *ADC2*, *SPDS1*, and *SPMS* [13]. On the other hand increase in ABA synthesis via Put accumulation; found to be upregulated in *ADC* overexpressing transgenic plants [95,96]. On the other hand, suppression of *ADC* gene (both *adc1* and *adc2*) resulted in the down-regulation of *NCED3* expression and hence reduced ABA-regulating genes expression. However, exogenous Put supplementation resulted in better tolerance to these plants under chilling stress [97].

Besides the regulatory role of Br as phytohormones in plant growth and physiological processes, it also confers abiotic stress tolerance singly or in keeping relations with ABA, AUX, CK, ET, JA, SA, and GA. A relationship between Br and PAs is recommended by the fact that epibrassinolide (EBL) treatment influences the level of PAs, and can alleviate Cu^2+^ stress [98]. Exogenous application of both EBL and Spd as cotreatment was more effective than their sole treatments to confer Cr tolerance in *R. sativus* [34]. On the other hand, Zn toxicity was fully conquered by the combined application of EBL and Spd [32]. Then, Mir et al. [32] recommended EBL and Spd as potential growth enhancers that promote biochemical parameters along with plant growth under Zn stress. Salicylic acid is regarded as a signal molecule to modulate defense mechanisms in plants from long before. Yet, the parallel alterations in SA and PAs contents of metal/metalloid(s) stressed affected plants have only been found in very few studies. However, some recent reports suggested that SA treatment influences PAs metabolism [99], but the exact cross-talk mechanisms under metal/metalloid(s) stress conditions are still elusive.

### 6.5. Polyamine Interacts with Ion Channels Modulate Metal/Metalloid(S) Stress Tolerance

Under metal/metalloid stress, the potential targets of ROS are the ion channels. Both H_2_O_2_ and OH^•^ modulate a number of ion channels, includes Ca^+^ influx and K^+^ efflux channels [100,101]. Moreover, PAs-induced NO generation influenced by K^+^ channel inhibition, stimulating H^+^-ATPase as well as PAs-activated Ca^2+^ efflux [101]. In addition, PAs actively regulate ion channel activity by membrane depolarization [71]; while PAs are also capable of blocking vacuolar cation channels (Spm^4+^> Spd ^3+^> Put^2+^). Thus, assist in vacuolar metal sequestration during stress [101].

Like metal/metalloid stress most of the abiotic stress responses share a variety of common elements, which are potential points of cross-talk. It is evident that PAs are the switching hub and interconnected at various levels. Among the biological signal transduction molecules H_2_O_2_ and NO are the main possible links between PAs and stress tolerance, yet, they are also interrelated. In addition, PAs are capable of influencing the ion channels, H_2_O_2_ and/or NO-modulated pathways, and the synthesis of plant hormones. This cross-talk between these factors together induce metal/metalloid(s) stress tolerance in plants.

## 7. Omics Approaches to Improve Polyamines Actions towards Metals/Metalloid(s) Action

Systems biology approaches are very useful for improving PAs biosynthesis and their action in the context of the broader genomic, metabolomic, proteomic and transcriptomic network. Not many analyses have been reported so far relating to the use of these approaches to improve PAs actions to enhance metal/metalloid(s) toxicity in plants, but still some interesting findings have been reported. Moreover, the genome sequences availability allows the use of these omics approaches to explore the variation of gene expression on a large genome scale. In this section, we have discussed the potential role of transcriptomics or transgenic approach and proteomics approaches to enhance PAs actions.

### 7.1. Transcriptomics

The accumulation of PAs is generally considered to be a common response of the plant to abiotic stresses, but the cause-effect relationship between the accumulation and protection of PAs remains unclear. To understand the PAs roles in stress tolerance, an effective strategy is to modulate their cellular levels, which has been realized using three different approaches—their exogenous application, PAs synthesis inhibitors use, and overexpression of their biosynthetic genes. Transcriptomics is a very useful approach to understanding PAs induced metal/metalloid(s) tolerance in plants. According to Alcázar et al. [102], studies based on transcriptomics showed different response and regulation of different genes involved in PAs biosynthesis and metabolism. Characterizing these genes can provide a better answer to ‘How we can improve PAs induced tolerance in plants in a better and sustainable way?’. Characterization of PAs mutants has provided evidence relating to the involvement of PAs in metal/metalloid(s) stress tolerance [103].

Different studies showed that overexpression of different PAs biosynthetic genes in metal/metalloid (s) sensitive plants was effective in improving stress tolerance (Table 3). The first step catalyzed by the *ADC* enzyme of PAs biosynthesis to generate Put from Arg. Under metal stress, a metabolic substrate such as *ADC* or *ODC* significantly reduced. Nonetheless, Urano et al. [18] showed that the lack of an *ADC* gene in an *Arabidopsis* mutant resulted in high sensitivity to stress conditions and showed reduced growth. Likewise, Wen et al. [104] reported that overexpressed *SPDS1* in European pear and noted that *SPDS1* played a very important role in enhancing Cu stress tolerance. Similar results have been reported under Cd, Zn, and Pb stress as well [56].

Polyamines accumulation under stress condition is mainly influenced by the de novo synthesis of PAs; however, their biosynthesis is primarily controlled and regulated at the transcriptional level. Therefore, understanding the expression pattern of PAs biosynthetic genes under stress conditions would be very useful in enhancing PAs production and their actions. Several types of research have been conducted to examine the steady-state transcriptional levels of PAs biosynthetic genes under different abiotic stress conditions [26] nonetheless under metal stress, hence limited information is available. Among different PAs biosynthetic genes, *SPDS*, *SPMS*, *SAMDC*, and *ADC* are important, and transcriptomics studies targeted these genes and showed a high level of tolerance [109,110]. Among them, *SPDS* and *ADC* have been widely studied under different abiotic stress in various plant species. For instance, overexpression of *ADC* genes in peach and eggplant up-regulated Cd tolerance [106,111], which is significantly correlated with high endogenous PAs levels and better antioxidant defense system. Other researchers similarly used transcriptomics approach and noted a high level of tolerance to different abiotic stress in *adc* eggplant transgenic lines [112,113]. Ornithine *δ*-aminotransferase (*δ*-OAT) is a pyridoxal-5′phosphate-dependent enzyme that has been proposed to be involved in the Pro and Arg metabolism. Overexpression of the *OsOAT* gene in rice showed considerably inhibited oxidative stress by triggering the antioxidant defense system [114]. These results are proving the multifaceted role of PAs in stress amelioration.

### 7.2. Proteomics

Proteomics is a very useful approach as it aimed at systematically sorting and characterizing complex protein structures or proteomes of the genome in different cells at different development stages. This approach also provides information relating to protein localization and protein interaction maps. From last two decades, numerous innovative methods such as protein detection using mass spectrometry, microarray technique, and the yeast two-hybrid system have been developed and used for large scale analysis of proteins, which involved in stress signaling and stress tolerance. Though several groups around the world have used the transcriptomics approach to investigate and to underpin expression level and patterns of different genes relating to PAs induced stress amelioration [115,116]; nonetheless, such analysis of mRNA levels has some limitations [117], such as the poor correlation between the expression patterns of mRNAs and their corresponding proteins [118]. Moreover, although the correlation between the expressions of mRNA and corresponding proteins has been identified [119] protein expression is also influenced or regulated at both translational and post-translational levels. Therefore, investigation and exploration of proteins at translational and post-translational levels can provide detailed insights into the response and functional interaction of proteins involved in PAs metabolism and their mode of action in improving metal/metalloid(s) tolerance in plants.

Although several studies, which revealed the use of proteomics approach in studying PAs induced metal/metalloid(s) stress tolerance, are scanty, published studies so far showed the promising role of proteomics in studying PAs induced metal/metalloid(s) stress tolerance. Wu et al. [120] used a proteomics approach and identified two proteins relating to SAM, which were involved in increasing Cd stress tolerance in *Solanum torvum*. Accumulation of SAM synthetase has also been noted in Medicago and rice root under Cd and As stress, respectively [121,122]. These findings have suggested that changes in the accumulation of SAM and subsequent biosynthesis of PAs caused metal/metalloid(s) stress tolerance [120,122]. Similarly, Yang et al. [123] also used a proteomics approach to study Cd-induced alteration in poplar plants to understand the molecular mechanisms behind Cd-induced toxicity. Under As stress, Ahsan et al. [121] employed a proteomics approach to study the role of proteins in rice root metabolism and found that most of the proteins were related to SAM. Tripathi et al. [116] reviewed and suggested that metal stress-induced ROS production and these ROS act as a signaling molecule and triggered the production of numerous proteins such as SAM, ADC or ODC and increase tolerance in plants. Moreover, targeting these proteins can be useful in improving metal stress in non-tolerant plant species. Similarly, Baig et al. [118] used proteomics approach and showed that Pb stress tolerance in soybean was associated with a high accumulation of thermospermine synthase ACAULIS5-like protein, this is encoded by ACAULIS5 (*ACL5*) gene and converts Spd to thermospermidine [124].

In another study, it was found that Cd tolerance in Cd-treated flax cell was associated with a high accumulation of expression of proteins and enzymes involved in PAs metabolism and SAM biosynthesis, thus high PAs protein levels resulted in high Cd tolerance. Brumbarova et al. [125] analyzed proteome changes in wild type and Fe mutant tomato plants treated with different levels of Fe-stress. They found that Fe mutants accumulated a high amount of non-functional proteins, which were transcription factors involved in Fe-induced changes in gene expression. Moreover, they also noted that tolerance capability of wild type tomato to Fe stress was associated with high expression of enzymes and proteins involved in ROS scavenging and SAM biosynthesis. Likewise, in rice, the concentration of As increased as a result of lipid peroxidation in an As-sensitive cultivar, while the As-tolerant cultivar exhibited high expression levels of SAM and GST, which were involved in improving tolerance to As [121]. Kim and Lee [126] employed proteomics approach and found similar results, and further suggested that targeting such proteins involved in PAs metabolism could be very useful in enhancing PAs induced metal/metalloid(s) tolerance in plants.

## 8. Conclusions

Metals/metalloid(s) toxicity harmfully affects plant growth and development, which results in global yield loss for agriculture. Moreover, toxic metals/metalloid(s) enter into the plant through the root and transferred to the upper parts, which subsequently come into the food chain and become a serious threat for a human being. Hence, it is essential to reduce metal/metalloid(s) from contaminated soil and find stress tolerant cultivars to cope with the future problem of nutritional food security. Polyamines including Put, Spd, and Spm play a significant role in physiology and biochemistry of plant, which confirm normal growth and development. Literature proves that enhanced PAs levels in plants have imperative functions in a wide range of physiological functions under abiotic stress including metals/metalloid(s) toxicity. Polyamines act as ROS scavengers, activate antioxidants, protect biomembranes and biomolecules, and provoke metal chelation under stress condition. In addition, PAs play a signaling role and interact with NO, different hormone, trace elements, and other signaling molecules for developing stress tolerance of the plant. As a result, exogenous applications of PAs getting popularity for enhancing metals/metalloid(s) stress tolerance. Nevertheless, the function of PAs metabolism and biosynthesis in plants for the metals/metalloid(s) stress tolerance has been just commenced to be recognized. Different omics approaches are also supportive to identify the contribution of PAs biosynthetic pathways in conferring stress tolerance. Countless efforts are needed to expose the molecular approaches of PAs induced protective role in stress tolerance. To date, many reports confirm the vital function of PAs under metals/metalloid(s) stress, but further research is required for a comprehensive study about the genes involved stress tolerance.

## Figures and Tables

**Table 1:** Polyamine-induced metal/metalloid(s) tolerance in different plant species.

Plant Species	Metal(s) Exposed	Changes in Endogenous PAs Level	Exogenous PAs Used	Tolerance	References
*Triticum aestivum* L.	1 mM CdCl_2_ for 5–15 d	-	0.1 mM Put, Spd or Spm pretreatment for 5 and 10 d	Lowered Cd-induced dehydration Increased chlorophyll content Confirmed better growth when pretreated for 10 days Restrain membrane biophysical properties under both Cd-stress	Benavides et al. [5]
*T. aestivum*	2 mM Cd as CdCl_2_	-	0.25 mM Spm, 0.50 mM Spd, and 1 mM Put as seed priming or as a foliar spray at 20, 40, and 60 DAS	Increased plant growth and grain yield Decreased Cd accumulation in the root, shoot, and leaf	Taie et al. [12]
*T. aestivum*	2 mM as PbCl_2_ for 45 d	-	0.25 mM Spm, 0.50 mM Spd, and 1 mM Put as seed priming or as a foliar spray at 20, 40, and 60 DAS	Increased plant growth and biomass accumulation Enhanced grain yield	Taie et al. [12]
*T. aestivum*	2 mM CdCl_2_ for 58 d	-	0.25 mM Spm, 0.50 mM Spd, or 1.0 mM put as seed priming and later foliar spraying	Enhanced growth and biomass Improved MSI and RWC and lowered EL Increased photosynthetic pigments contents Improved nutritional status Increased Pro content Improved water use efficiency (WUE) Enhanced yield contributing attributes and yield Reduced Cd accumulation by roots, shoots, and grain	Rady et al. [28]
*T. aestivum*	1 mM CuCl_2_ for 5–15 d	-	0.1 mM Put, Spd, or Spm pretreatment for 5 and 10 d	Improved growth under Cu-stress, which was better after 10 days of pretreatment. Put stimulated chlorophyll content and controlled membrane damage under Cu stress	Benavides et al. [5]
*T. aestivum*	1 mM Cd as CdCl_2_	Increased PAs content	2 mM Spd or 2 mM Spm as a seed treatment for 6 h	Increased growth and biomass Increased RWC, while decrease Pro content Decrease electrolyte leakage (EL) and increase membrane stability index (MSI) Increased protein and, starch content	Rady and Hemida [11]
*T. aestivum*	30 µM AlCl_3_	Increased Spd	Put, 2 mM	Increased Spd content Decrease cell death in both genotypes	Yu et al. [29]
*T. aestivum*	2 mM Cd	Increased Spm and Spd content	-	Increased Pro content	Howladar et al. [30]
*T. aestivum*	2.0 mM Pb^2+^	-	0.25 mM Spm, 0.50 mM Spd or 1.0 mM Put as seed treatment	Improved plant height, leaf number, and fresh biomass Increased total Chl and car content with higher RWC Exhibit higher yield contributing characters and produced a higher yield Enhanced plant macronutrient (N, K, and P) content	Rady et al. [31]
*Helianthus annuus* L.	1 mM CdCl_2_ for 5–15 d	Increased Put, Spd, and Spm content	0.1 mM Put, Spd, or Spm pretreatment for 5 and 10 d	Put enhanced chlorophyll content Pretreatment for 10 days showed better tolerance Inhibited the negative effect of Cd on membrane biophysical properties	Benavides et al. [5]
*H. annuus*	1 mM CuCl_2_ for 5–15 d	Increased Put, Spd, and Spm content	0.1 mM Put, Spd, or Spm pretreatment for 5 and 10 d	Put and Spm stimulated chlorophyll content in 10 days of pretreatment showed better plant growth Inhibited the negative effect of Cu on plant membrane integrity	Benavides et al. [5]
*Vigna radiata* L.	1.5 mM CdCl_2_	Increased Spd and Spm content with decrease the Put/PAs ratio	Put, 0.2 mM	Increased Pro and NO content Restored the growth inhibition under Cd exposure Increased the RWC, succulence and Chl content Decreased Cd accumulation in both roots and shoots	Nahar et al. [1]
*V. radiata*	Zn, 200 mg kg^−1^ soil as ZnSO_4_·7H_2_O	-	1.0 mM Spd, foliar application	Increased photosynthetic pigments, stomatal conductance, and intercellular CO_2_ concentration Increased plant growth, biomass, leaf area, and leaf water potential	Mir et al. [32]
*Chlorella vulgaris* Beijerinck	100 µM of Cu as Cu(NO_3_)_2_·3H_2_O	-	100 μM Spd	Increase cell number indicated growth stimulation Increased Chl *a*, Chl *b* and car content	Piotrowska-Niczyporuk et al. [33]
*C. vulgaris*	100 µM Cd, as Cd(NO_3_)_2_·4H_2_O	-	100 μM Spd	Stimulated growth Increased photosynthetic pigments (Chl *a*, Chl *b* and car)	Piotrowska-Niczyporuk et al. [33]
*C. vulgaris*	100 µM of Pb Pb(NO_3_)_2_	-	100 μM Spd	Stimulated growth Increased photosynthetic pigments	Piotrowska-Niczyporuk et al. [33]
*Raphanus sativus* L.	1.2 mM Cr as (K_2_CrO_4_)	Increased Put and Spd content	1 mM Spd as cotreatment	Increased growth and biomass and total soluble sugar Increased photosynthetic pigments content and PSII quantum yield	Choudhary et al. [34]
*Poncirus trifoliata* L.	0, 0.25, 0.50, 0.75, 1.0, 1.25 mM Cr as K_2_Cr_2_O_7_	Increased PAs content	-	Enhanced PAs degradation enzymes (PAO and DAO) activities Increased PS conjugated PAs, and PIS-bound PAs improved growth and development	Shahid et al. [27]
*Citrus reshni* L.	0, 0.25, 0.50, 0.75, 1.0, 1.25 mM Cr as K_2_Cr_2_O_7_	Increased PAs content	-	Enhanced PAO and DAO activities Increased PS PAs and PIS PAs Improve growth and development	Shahid et al. [27]
*C. limonia* Osbeck (CL)	0, 0.25, 0.50, 0.75, 1.0, 1.25 mM Cr as K_2_Cr_2_O_7_	Increased PAs content	-	Upregulated PAO and DAO enzyme activities Increased PS PAs and PIS PAs Growth and development enhanced	Shahid et al. [27]
*Salix matsudana* Koidz.	0.05 and 0.10 mM Cd	Increased endogenous Spd and Put contents	0.25 mM Spd as cotreatment for 3 d	Enhanced shoot and root growth Increased mineral contents i.e., Cu, Zn, and Fe Increased BCF and TF of Cd in root and cutting Decreased BCF and TF in twig and leaf Increased endogenous NO content	Tang et al. [35]
*Potamogeton crispus* L.	30, 50, 70 µM Cd as CdCl_2_	Increased Put, PS Put, and PIS Put content	-	Membrane stabilization Increased PAO and DAO activity	Yang et al. [36]

**Table 2:** Polyamine mediated antioxidant defense in different plant species under metal/metalloid stresses.

Crop Species	Metal Exposure	Changes in Endogenous PAs Level	Exogenous PAs Applications	Antioxidant Defense System	References
*Triticum aestivum* L.	2 mM Cd as CdCl_2_ and 2 mM as PbCl_2_ for 45 d	-	0.25 mM Spm, 0.50 mM Spd, and 1 mM Put as seed priming or as a foliar spray at 20, 40, and 60 DAS	Enhanced the activity of SOD, CAT, POD, and GR Improved ascorbic acid oxidase (AAO), and polyphenol oxidase (PPO) activities	Taie et al. [12]
*T. aestivum*	2 mM as PbCl_2_ for 45 d	-	0.25 mM Spm, 0.50 mM Spd, and 1 mM Put as seed priming or as a foliar spray at 20, 40 and 60 DAS	Enhanced the activity of SOD, CAT, POD, GR Improved ascorbic acid oxidase (AAO), and polyphenol oxidase (PPO) activities	Taie et al. [12]
*T. aestivum*	2 mM CdCl_2_ for 58 d	-	0.25 mM Spm, 0.50 mM Spd, 1.0 mM Put as seed priming and later foliar spraying	Increased POD and CAT activity Decreased SOD activity	Rady et al. [28]
*T. aestivum*	30 µM AlCl_3_	Increased Spd	Put, 2 mM as cotreatment	Inhibited plasma membrane NADPH oxidase and CW-PAO activity in Al-stressed wheat and thus reduced H_2_O_2_ accumulation Decreased SOD, CAT, POD, APX, GR, GST activities Decreased LOX activity and Thiobarbituric acid (TBARS) content	Yu et al. [29]
*T. aestivum*	2 mM Cd	Increased endogenous Spm and Spd upon Cd exposure	-	Increased AsA, GSH content with higher SOD, CAT, POD activity	Howladar et al. [30]
*T. aestivum*	1 mM Cd as CdCl_2_	Increased PAs content by pretreated seedlings under stress affection	2 mM Spd or 2 mM Spm as a seed treatment for 6 h	Increased AsA and GSH content Enhanced SOD and CAT activity Decreased POX and APX activity Lowered H_2_O_2_ and MDA generation	Rady and Hemida [11]
*T. aestivum*	0.5 mM Cd as CdCl_2_	Increased PAs content such as Put, Spd, and Spm	0.5 and 1.0 mM Spm	Decreased ADC and ODC with lowered DAO activities Lowered SOD, GPX, with increased of GR Increased APX activity under Cd stressed the plant Increased GSH content under Cd stress while decreased at Cu treated plant	Groppa et al. [37]
*T. aestivum*	0.5 mM Cu as CuCl_2_	-	0.5 and 1.0 mM Spm	Decreased ADC and ODC with lowered DAO activities Lowered SOD, GPX, with increased of GR Increased APX activity under Cd stressed the plant Increased GSH content under Cd stress while decreased at Cu treated plant	Groppa et al. [37]
*Oryza sativa* L.	5 mM CdCl_2_	-	5 mM Put, 5 mM Spd, and 5 mM Spm	Increased protein content under Cd exposure Increased AsA and GSH content at Cd stressed rice Decreased SOD, CAT, APX, GR and POX activity Reduced H_2_O_2_ and MDA content significantly	Hsu and Kao [38]
*Vigna radiata*	1.5 mMCdCl_2_	Increased Spd and Spd content with decrease the Put/PAs ratio	Put, 0.2 mM, as pretreatment for 24 h	Boosted up AsA and GSH content, while DHA content was decreased Increases AsA/DHA ratio and decreased GSH/GSSG ratio Increased SOD, CAT, APX, MDHAR, DHAR and GST activities Decreased the ROS generation with lower malondialdehyde (MDA) and lipoxygenase (LOX) activity Improved the glyoxalase system by increasing Gly II activity with lowered MG content	Nahar et al. [1]
*V. radiata*	200 mg kg^−1^ soil as ZnSO_4_·7H_2_O	-	1.0 mM Spd, foliar application	Increased SOD, CAT and POX activity	Mir et al. [32]
*Chlorella vulgaris* Beijerinck	100 µM of Cd as Cd(NO_3_)_2_·4H_2_O	-	100 μM Spd	Increased AsA and GSH accumulation Increased SOD, CAT, and APX activity Decreased H_2_O_2_ and MDA generation	Piotrowska-Niczyporuk et al. [33]
*C. vulgaris*	100 µM Pb as, Pb(NO_3_)_2_	-	100 μM Spd	Increased AsA and GSH accumulation Increased SOD, CAT, and APX activity Decreased H_2_O_2_ and MDA generation	Piotrowska-Niczyporuk et al. [33]
*C. vulgaris*	100 µM Cu as Cu(NO_3_)_2_·3H_2_O	-	100 μM Spd	Increased AsA and GSH accumulation Increased SOD, CAT, and APX activity Decreased H_2_O_2_ and MDA generation	Piotrowska-Niczyporuk et al. [33]
*Raphanus sativus*	1.2 mM Cr as (K_2_CrO_4_)	Increased Put and Spd content	1 mM Spd as cotreatment	Increased GSH, AsA, contents Enhanced accumulation of osmolyte (Pro, GB, and Phenol) Increased SOD, and GR activity where decrease Cat and POD activities in Cr-stressed plants Lowered NADPH oxidase activity Decreased MDA and H_2_O_2_ production	Choudhary et al. [34]
*Salix matsudana* Koidz.	0.05 and 0.10 mM Cd	Increased endogenous Spd and Put contents	0.25 mM Spd as cotreatment for 3 d	Upregulated SOD, CAT, GR GPX, and APX activities Enhanced AsA and GSH contents Increased endogenous NO content Decreased MDA, O_2_^•−^, and H_2_O_2_ production	Tang et al. [35]
*Helianthus annuus*	0.5 mM Cd as CdCl_2_	Increased endogenous Put and Spd levels	1.0 mM Spd and Spm	Increased APX and GR activity under Cd stress while reducing the SOD activity Enhanced the SOD and GR activity with lower APX activity under Cu stressed plant	Groppa et al. [39]
*H. annuus*	0.5 mM Cu as CuCl_2_	Increased endogenous Put and Spd levels	1.0 mM Spd and Spm	Increased APX and GR activity under Cd stress while reducing the SOD activity Enhanced the SOD and GR activity with lower APX activity under Cu stressed plant	Groppa et al. [39]

**Table 3:** List of transgenic plants encoding PA biosynthetic genes exhibiting high heavy metal tolerance.

PA gene	Host plant/organism	Transgenic plant	Targeted metals (tolerance)	Reference
*SPDS 1*	Apple	European Pear	Cd, Zn, and Pb	Wen et al. [56]
*SPDS 1*	Apple	European Pear	Cu stress	Wen et al. [104]
*SPDS 1*	Apple	European Pear	Cd stress	Wen et al. [105]
*ADC* gene	*Agrobacterium*	Eggplant	Cd stress	Prabhavathi and Rajam [106]
*SPDS 1*	Apple	European Pear	Al stress	Wen et al. [107]
*ALD*		*Arabidopsis*	Cd and Cu stress	Sunkar et al. [108]

## Abbreviations

AAO	Ascorbic acid oxidase
ACC synthase	1-aminocyclopropane-1-carboxylic-acid synthase
ACS	1-aminocyclopropane-1-carboxylic-acid synthase
ACC	1-aminocyclopropane-1-carboxylic-acid
ABAL	4-aminobutanal
ABA	Abscisic acid
ATP	Adenosine triphosphate
AIH	Agmatin iminohydrolase
ADC	Arginine decarboxylase
APX	Ascorbate peroxidase
AsA	Ascorbate
AUX	Auxins
BAC	Biological accumulation coefficient
BCF	Biological concentration factor
Br	Brassinosteroids
CAT	Catalase
CW-PAO	Cell wall-PAO
CK	Cytokinins
dcSAM	Decarboxylated *S*-adenosylmethionine
DHAR	Dehydroascorbate reductase
DAO	Diamine oxidase
EL	Electrolyte leakage
EBL	Epibrassinolide
ET	Ethylene
GA	Gibberellins
GABA	γ-aminobutyric acid
GPX	Glutathione peroxidase
GR	Glutathione reductase
GST	Glutathione *S*-transferase
GSH	Glutathione
GB	Glycine betaine
Gly I	Glyoxalase I
Gly II	Glyoxalase II
IAA	Indole acetic acid
JA	Jasmonic acids
LOX	Lipoxygenase
MDA	Malondialdehyde
MSI	Membrane stability index
MTs	Metallothioneins
MG	Methylglyoxal
MAPKs	Mitogen-activated protein kinases
MDHAR	Monodehydroascorbate reductase
CPA	N-carbamoylputrescine amidohydrolase
NADPH	Nicotinamide adenine dinucleotide phosphate
NR	Nitrate reductase
NOS	NO synthase
OTC	Ornithine transcarbamoylase
*δ*-OAT	Ornithine *δ*-aminotransferase
ODC	Ortinine decarboxylase
PC	Phytochelatins
PAs	Polyamines
PPO	Polyphenol oxidase
Pro	Proline
Put	Putrescine
PDH	Pyrroline dehydrogenase
ROS	Reactive oxygen species
RWC	Relative water content
SAMDC	*S*-adenosylmethionine decarboxylase
SAM	*S*-adenosylmethionine
SA	Salicylic acid
SNP	Sodium nitroprusside
SPDS	Spermidine synthase
Spd	Spermidine
SPMS	Spermine synthase
Spm	Spermine
SOD	Superoxide dismutase
TBARS	Thiobarbituric acid
TF	Translocation factor
